# Associations in Perceived Health and Persistent Breathlessness: A Cross-Sectional Study

**DOI:** 10.1089/pmr.2022.0071

**Published:** 2023-04-20

**Authors:** Slavica Kochovska, Sungwon Chang, Max Olsson, Magnus Ekström, David C. Currow

**Affiliations:** ^1^ Faculty of Science, Medicine and Health, University of Wollongong, Wollongong, New South Wales, Australia.; ^2^ IMPACCT, Faculty of Health, University of Technology Sydney, Sydney, New South Wales, Australia.; ^3^ Department of Clinical Sciences Lund, Respiratory Medicine and Allergology, Faculty of Medicine, Lund University, Lund, Sweden.

**Keywords:** breathlessness, dyspnea, global impression of change, palliative care, perceived health, population studies

## Abstract

**Background:**

Persistent breathlessness is debilitating and increases in prevalence with advanced age and at end of life. This study aimed to evaluate any relationship between self-reported global impressions of change (GIC) in perceived health and breathlessness in older men.

**Design:**

Cross-sectional study of 73-year-old Swedish men in the VAScular and Chronic Obstructive Lung disease study. A postal survey included items on perceived changes in health and breathlessness (GIC scales) and breathlessness (assessed using the modified Medical Research Council [mMRC] breathlessness scale, Dyspnea-12 and Multidimensional Dyspnea Scale) since age 65.

**Results:**

Of 801 respondents, breathlessness (mMRC ≥2) was reported by 17.9%, worsening breathlessness by 29.1%, and worsening perceived health by 51.3%. Worsening breathlessness was strongly correlated with worsening perceived health (Pearson's correlation coefficient of 0.68 [*p* < 0.001] and Kendall's τ of 0.56 [*p* < 0.001]) and associated with more limited function (47.2% vs. 29.7%; *p* < 0.0001) and increased rates of anxiety/depression.

**Conclusion:**

The strong correlation between perceived changes in health and persistent breathlessness helps delineate a more comprehensive picture of the challenges faced by older adults living with this disabling symptom.

## Introduction

Persistent (chronic) breathlessness is a disabling syndrome^1,2^ that becomes more prevalent as people age and in people with life-limiting illnesses, often worsening in the last weeks and days of life.^3^ Persistent breathlessness impairs the person's quality of life^4^ and, as it worsens, increasingly affects their physical and psychosocial well-being,^5^ causing marked disability.^6^ The symptom is associated with increased health service utilization and its presence is a strong predictor of mortality.^7,8^ Its prevalence in people with life-limiting illnesses is also indicative of palliative care needs.^9,10^

Older people living with chronic progressive conditions have greater symptom burden, including breathlessness.^11–13^ This has implications for their overall health and well-being, as well as the provision of adequate symptom management to optimize their quality of life. People with chronic progressive conditions live with breathlessness for long periods of time,^14^ often reducing exertion and physical activity as a means of helping to manage their breathlessness. This process may trigger a vicious cycle of deconditioning, which results in more debilitating breathlessness.^15^ Given the symptom's often progressive trajectory, it is important to understand how changes in persistent breathlessness relate to changes in perceived overall health, especially in older individuals.^16^ A deeper understanding of any such relationship could help generate the introduction of more timely symptom interventions, tailored to anticipate changing personal needs and preferences, including in the palliative care setting.

This study aimed to evaluate any association between self-reported global impressions of change (GIC) in health and the respondent's self-rated GIC of breathlessness in a large population sample of elderly men.

## Methods

### Design and population

We conducted a cross-sectional analysis of data reported by 73-year-old men in Blekinge, Sweden enrolled in the VAScular and Chronic Obstructive Lung disease (VASCOL) study.^17^ Recruitment of participants in the VASCOL study was through a screening program conducted in 2010 to 2012 for aortic aneurysms of 65-year-old men (where of the 1900 invited, 1302 participated in VASCOL). The data for this study were collected at follow-up in 2019. A postal survey with questions on perceived health and breathlessness was sent to live participants with a known address. Of 1193 men invited, 907 (76%) completed the survey. Only completed answers for GIC health and GIC breathlessness were included in this analysis. VASCOL study details have been presented previously.^17^

### Ethical considerations

The study was approved by the Swedish Ethical Review Authority (2019-00134), and written informed consent obtained from participants.

### Assessments

Self-reported questionnaire data included height, weight, and physician-diagnosed respiratory and cardiovascular diseases.

Change in perceived health (as a global concept for each respondent and used widely in measuring perceived changes in health states) and breathlessness were measured with the GIC, a 7-point ordinal scale referring to respondents' assessment in changes over the previous seven years (since age 65): *very much better* (1), *much better* (2), *minimally better* (3), *no difference* (4), *minimally worse* (5), *much worse* (6), or *very much worse* (7).^18^ GIC scores were dichotomized for the analysis with 1 to 4 indicating “improvement or no change” and 5 to 7 indicating “worsening.”

The presence and intensity of breathlessness (in the last two weeks) were measured further using the unidimensional modified Medical Research Council (mMRC) breathlessness scale^19^ and the multidimensional Dyspnea-12 (D-12)^20,21^ and Multidimensional Dyspnea profile (MDP).^21,22^ The mMRC^19^ is an ordinal scale that reflects the level of exertion before being limited by breathlessness. The D-12 consists of 12 items comprised within two subdomains: *physical* and *affective*. The minimum clinically important difference (MCID) for the total score of 36 has been reported as 2.83 (95% confidence interval [CI] 1.99–3.66).^23^ The MDP^21,22^ consists of 11 items within three subdomains: *A1 unpleasantness*, *immediate perception*, and *emotional response*. The MCID for the A1 component (range 0–10) has been reported as 0.82 (95% CI 0.56–1.08).^23^ Respondents who reported any breathlessness were also asked about its duration (*number of years, less than one year,* or *I don´t remember*).

For the period of the previous two weeks, respondents were also asked, using validated measures, about anxiety/depression and functional status. The Hospital Anxiety and Depression Scale was used to assess anxiety and depression (each symptom ranges 0–21; higher scores indicating more severe symptom)^24^ and the World Health Organization Performance Status scale was used to assess respondents' functional status (scores 0–4; higher scores indicating impaired function).^25^

### Statistical analyses

Differences in the participants' characteristics were analyzed using *t* tests, chi square, and Mann–Whitney *U* tests as appropriate. The primary outcome was the correlation between self-reported changes in health scores and persistent breathlessness, assessed using Kendall's tau and Pearson's correlation coefficient. No data were imputed. Statistical significance was defined as a two-tailed *p*-value <0.05. Statistical analyses were conducted using SPSS for Windows version 26.0 (SPSS, Chicago, IL; 2011).

## Results

Of 1193 men contactable, 801 (67.1%) were analyzed (Table 1). Characteristics of those included and not included in this study were similar (data not shown). Worsening health was reported by 411 (51.3%) respondents. Worsening breathlessness was reported by 233 (29.1%) respondents. Breathlessness was reported by 8.5% as mMRC 2, 4.6% as mMRC 3 and 4.8% as mMRC 4, with a median duration of breathlessness of two years (interquartile range 0.0, 5.0) (Table 1). Self-reported cardiovascular and respiratory diseases were more prevalent in those reporting greater GIC than those who did not. Overweight or obesity was present in 70.6% of respondents.

**Table 1.** Global Impression of Change in Breathlessness and Health Dichotomized into Two Response Categories (*Better* or *No Different*; and *Worse*)

	Total (***n*** = 801)	GIC					
		Breathlessness			Health		
		Better or no difference, scores 1–4 (***n*** = 568)	Worse, scores 5–7 (***n*** = 233)	***p***	Better or no difference, scores 1–4 (***n*** = 390)	Worse, scores 5–7 (***n*** = 411)	***p***
WHOPS measure^a^				<0.001			<0.001
Fully active; no performance restrictions (0)	572 (72.3)	451 (80.2)	121 (52.8)		334 (86.1)	238 (59.1)	
Strenuous physical activity restricted; fully ambulatory and able to carry out light work (1)	175 (22.1)	89 (15.8)	86 (37.6)		39 (10.1)	136 (33.7)	
Worse than capable of all self-care but unable to carry out any work activities. Up and about >50% of waking hours (2–4)	44 (5.6)	22 (3.9)	22 (9.6)		15 (3.9)	29 (7.2)	
HADS anxiety^a^				<0.001			<0.001
Normal (0–7)	671 (87.0)	492 (90.6)	179 (78.5)		351 (93.4)	320 (81.0)	
Borderline (8–10)	58 (7.5)	33 (6.1)	25 (11.0)		17 (4.5)	41 (10.4)	
Abnormal (11–21)	42 (5.4)	18 (3.3)	24 (10.5)		8 (2.1)	34 (8.6)	
HADS depression				<0.001			<0.001
Normal (0–7)	713 (91.6)	521 (94.9)	192 (83.8)		364 (96.6)	349 (87.0)	
Borderline (8–10)	44 (5.7)	22 (4.0)	22 (9.6)		11 (2.9)	33 (8.2)	
Abnormal (11–21)	21 (2.7)	6 (1.1)	15 (6.6)		2 (0.5)	19 (4.7)	
Self-reported cardiac conditions							
Heart attack	74 (9.2)	43 (7.6)	31 (13.3)	0.010	28 (7.2)	46 (11.2)	0.050
Angina	57 (7.1)	37 (6.5)	20 (8.6)	0.300	24 (6.2)	33 (8.0)	0.300
Heart failure	30 (3.7)	18 (3.2)	12 (5.2)	0.200	12 (3.1)	18 (4.4)	0.300
Valvular heart failure	40 (5.0)	24 (4.2)	16 (6.9)	0.100	19 (4.9)	21 (5.1)	0.900
Stroke	57 (7.1)	35 (6.2)	22 (9.4)	0.100	21 (5.4)	36 (8.8)	0.060
High blood pressure	439 (54.8)	294 (51.8)	145 (62.2)	0.010	192 (49.2)	247 (60.1)	0.002
High cholesterol	212 (26.5)	142 (25.0)	70 (30.0)	0.100	93 (23.8)	119 (29.0)	0.100
Self-reported respiratory conditions							
COPD	31 (3.9)	15 (2.6)	16 (6.9)	0.005	10 (2.6)	21 (5.1)	0.060
Asthma	40 (5.0)	20 (3.5)	20 (8.6)	0.003	15 (3.8)	25 (6.1)	0.100
Other lung disease	9 (1.1)	6 (1.1)	3 (1.3)	0.800	6 (1.5)	3 (0.7)	0.300
Tuberculosis	3 (0.4)	1 (0.2)	2 (0.9)	0.200	1 (0.3)	2 (0.5)	0.600
Measures of breathlessness							
Dyspnea-12 total score							
Mean (SD)	1.6 (4.1)	0.7 (2.2)	4.0 (6.1)	<0.001^a^	0.5 (1.8)	2.7 (5.2)	<0.001^a^
Mean (SD)							
MDP-A1	0.7 (1.4)	0.3 (0.8)	3.1 (1.8)	<0.001^a^	0.3 (0.8)	1.1 (1.7)	<0.001^a^
mMRC breathlessness scale							
*n* (%)				<0.001			<0.001
0	532 (67.9)	445 (79.5)	87 (38.8)		318 (82.4)	214 (53.8)	
1	111 (14.2)	63 (11.3)	48 (21.4)		34 (8.8)	77 (19.3)	
2	67 (8.5)	24 (4.3)	43 (19.2)		17 (4.4)	50 (12.6)	
3	36 (4.6)	15 (2.7)	21 (9.4)		8 (2.1)	28 (7.0)	
4	38 (4.8)	13 (2.3)	25 (11.2)		9 (2.3)	29 (7.3)	

^a^ Mann–Whitney test.

COPD, chronic obstructive pulmonary disease; GIC, global impression of change; HADS, Hospital Anxiety and Depression Scale; MDP-A1, Multidimensional Dyspnea Profile (affective component); mMRC, modified Medical Research Council; SD, standard deviation; WHOPS, World Health Organization Performance Status.

In people with improvement or no change in breathlessness since age 65, 80.2% reported no restrictions on function, whereas for people with worsening breathlessness, only 52.8% reported no restrictions on function (*p* < 0.0001). *Current breathlessness* on the D-12 scores were 0.7 (standard deviation [SD] 2.2) and 4.0 (SD 6.1) for the groups, respectively. Worsening breathlessness was related to increased rates of anxiety and depression (Table 1).

**FIG. 1.**
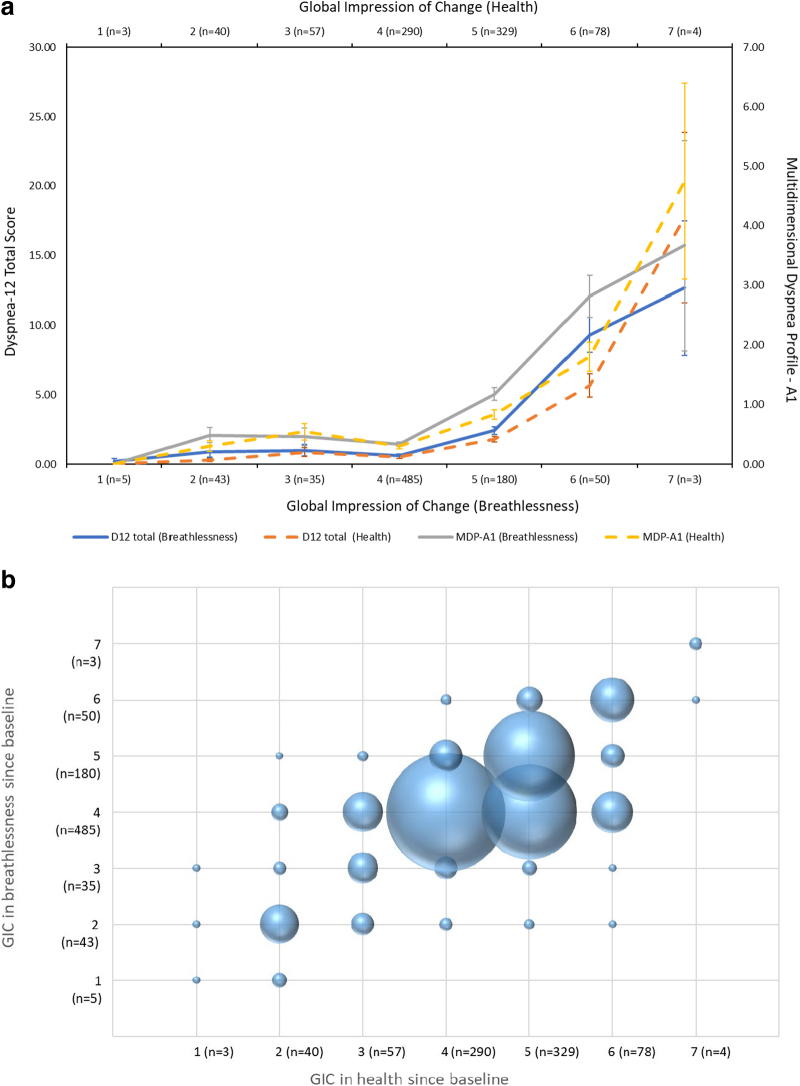
**(a)** Breathlessness in the last two weeks (D-12 Total and MDP-A1 scores) as functions of GIC in health and breathlessness since age 65, with standard error lines. **(b)** The relationship between GIC for health status and persistent breathlessness in 801 Swedish men aged 73 with the size of each sphere proportional to the size of that group of respondents. D-12, Dyspnea-12; GIC, global impression of change; MDP-A1, Multidimensional Dyspnea Profile (affective component).

There was a strong correlation between worsening perceived health and perceived breathlessness (Pearson's correlation coefficient of 0.68 [*p* < 0.001]; Kendall's τ of 0.56 [*p* < 0.001]). When plotted against D-12 and MDP total scores, respectively, the relationship of self-reported worsening health with increased breathlessness scores are seen (Fig. 1a). This strong correlation is also seen in changes in health and breathlessness statuses (Fig. 1b).

## Discussion

This study demonstrates a strong relationship between self-reported worsening of health status and worsening persistent breathlessness over seven years, reflecting other population studies,^26^ including a comparable age group.^27^ The negative impact of breathlessness on well-being is greater as breathlessness intensifies, which also increasingly limits function.

By demonstrating links between self-perceived health and persistent breathlessness, this study complements findings from the same population showing that persistent breathlessness is associated with fewer physical, social, and sexual activities.^28^ The studies demonstrate different associations between a person's well-being and the presence of persistent breathlessness. This study shows that the limitations associated with the symptom are particularly felt by people with self-reported cardiovascular and respiratory conditions.

This aligns with findings reported for an Irish population of similar age (mean age 73; 54% men) where living with chronic conditions meant living with high symptom burden (including breathlessness) and unmet needs.^11,12^ Despite this, persistent breathlessness is often under-recognized and undertreated^29^ because of clinician and patient factors. Given that the increase in symptom severity over time is likely to be even more burdensome for older people with comorbidities and coexisting symptoms, identifying the presence and severity of persistent breathlessness across the disease/symptom trajectory is critically important as its timely and effective management may improve overall well-being.

The findings here are consistent with the aspects of well-being and breathlessness that have shown population-level associations, including quality of life, anxiety and depression, functional status, and specific areas of personhood such as level of sexual activity.^5,27,30^ This study further highlights these impacts in older men.

The study adds an additional dimension of the associations with persistent breathlessness on the well-being of older adults: that a self-reported health status is perceived to worsen as the symptom worsens. The strong correlation between perceived changes in health and persistent breathlessness helps delineate a more comprehensive picture of the challenges faced by older adults living with this debilitating symptom.

This is a large population sample, but it is of males at one age, which limits the generalizability of the findings for women and individuals of other ages. The changes in health and breathlessness were based on recall by the participants and not measured longitudinally. There is a risk that participants were recalling the final or most intense experience of a symptom (“peak-end-rule”),^31^ which could have led to an overestimate of breathlessness in this study. The risk of any cognitive impairment on recall could also not be evaluated. Data to date suggest that mild to moderate impairment is not limiting to people's capacity to report symptoms,^32^ but the role of cognitive impairment on symptom reporting more broadly has not been widely researched and should be an area of future inquiry. Future research should also validate these findings in more diverse populations, longitudinally.

## Abbreviations Used

CI: confidence interval
COPD: chronic obstructive pulmonary disease
D-12: Dyspnea-12
GIC: global impressions of change
HADS: Hospital Anxiety and Depression Scale
MCID: minimum clinically important difference
MDP-A1: Multidimensional Dyspnea Profile (affective component)
MDP: Multidimensional Dyspnea profile
mMRC: modified Medical Research Council
SD: standard deviation
VASCOL: VAScular and Chronic Obstructive Lung disease
WHOPS: World Health Organization Performance Status
